# Prophylactic Activated Recombinant Factor VII in Liver Resection and Liver Transplantation: Systematic Review and Meta-Analysis

**DOI:** 10.1371/journal.pone.0022581

**Published:** 2011-07-27

**Authors:** Norberto C. Chavez-Tapia, Roberto Alfaro-Lara, Felix Tellez-Avila, Tonatiuh Barrientos-Gutiérrez, Octavio González-Chon, Nahum Mendez-Sanchez, Misael Uribe

**Affiliations:** 1 Obesity and Digestive Diseases Unit, Medica Sur Clinic and Foundation, Mexico City, Mexico; 2 Endoscopy Department, Instituto Nacional de Ciencias Médicas y Nutrición Salvador Zubirán, Mexico City Mexico; 3 National Institute of Public Health. Mexico City, Mexico; 4 Cardiology Department, Medica Sur Clinic and Foundation, Mexico City, Mexico; University of Colorado Denver, United States of America

## Abstract

**Background and Aim:**

Intraoperative blood loss is a frequent complication of hepatic resection and orthotopic liver transplantation. Recombinant activated coagulation factor VII (rFVIIa) is a coagulation protein that induces hemostasis by directly activating factor X. There is no clear information about the prophylactic value of rFVIIa in hepatobiliary surgery, specifically in liver resection and orthotopic liver transplantation. The aim of this study was to assess the effect of rFVIIa prophylaxis to prevent mortality and bleeding resulting from hepatobiliary surgery.

**Methods:**

Relevant randomized trials were identified by searching The Cochrane Central Register of Controlled Trials in The Cochrane Library, MEDLINE, EMBASE, and Science Citation Index. Randomized clinical trials comparing different rFVIIa prophylactic schemas against placebo or no intervention to prevent bleeding in hepatobiliary surgery were included. Adults undergoing liver resection, partial hepatectomy, or orthotopic liver transplantation were included. Dichotomous data were analyzed calculating odds ratios (ORs) and 95% confidence intervals (CIs). Continuous data were analyzed calculating mean differences (MD) and 95% CIs.

**Results:**

Four randomized controlled trials were included. There were no significant differences between rFVIIa and placebo for mortality (OR 0.96; 95% CI 0.35–2.62), red blood cell units (MD 0.32; 95% CI −0.08–0.72) or adverse events (OR 1.55; 95% CI 0.97–2.49).

**Conclusions:**

The available information is limited, precluding the ability to draw conclusions regarding bleeding prophylaxis in hepatobiliary surgery using rFVIIa. Although an apparent lack of effect was observed in all outcomes studied, further research is needed.

## Introduction

Hepatic resection is often accompanied by intraoperative blood loss occurring during parenchyma transection or tumor resection. Similarly, orthotopic liver transplantation (OLT) may also cause excessive blood loss during surgery, which may lead to increased postoperative morbidity and mortality [1]. Many surgical techniques to achieve vascular control during hepatic resection have been proposed, producing good results [2]. Recently, pharmacological approaches have been used to induce a primary hemostatic effect in an attempt to reduce intraoperative blood loss [3].

Recombinant activated coagulation factor VII (rFVIIa) (NovoSeven, Novo Nordisk, Denmark) is a coagulation protein that induces hemostasis through direct activation of factor X, starting the conversion of prothrombin to thrombin to form a hemostatic clot. At the site of vascular injury, rFVIIa binds to the surface of activated platelets, increasing localized thrombin generation [4]. Use of rFVIIa has been approved for the treatment of bleeding in patients with hemophilia A and B, acquired hemophilia, factor VII deficiency, or Glanzmann thrombasthenia refractory to platelet administration. It has also been used to manage hemorrhage caused by severe traumatic injury or transplantation, cardiac surgery, treatment of intracerebral hemorrhage, and bleeding associated with anticoagulation therapy [5], [6].

No clear information exists regarding the use of rFVIIa to prevent bleeding in hepatobiliary surgery. The present study aimed to assess the effect of prophylactic rFVIIa in preventing bleeding and reducing mortality in patients undergoing partial hepatectomy or OLT.

## Methods

### Types of Studies

Randomized clinical trials that compared different rFVIIa prophylactic schedules and dosages against placebo or no intervention to prevent bleeding in hepatobiliary surgery were included. These trials were included irrespective of publication status, language, and blinding.

### Types of Participants

Patients were included if they were 18 years or older and had undergone liver resection, partial hepatectomy, or OLT, irrespective of the indication for the surgical procedure and liver functional status assessed by any scale. Patients with recent abdominal surgery or bleeding disorders and those scheduled for multiorgan transplantation were excluded.

### Types of Interventions

The following interventions were considered:

use, dosage, benefits, and harms of prophylactic rFVIIa in OLT;
use, dosage, benefits, and harms of prophylactic rFVIIa in liver resection.

Control groups received placebo or no intervention.

### Types of Outcome Measures

The primary outcome measures were:

mortality rate;
transfusion requirements;
adverse events, which included:
  any serious adverse events that were fatal, life threatening, or requiring inpatient hospitalization or prolongation of existing hospitalization;
  any adverse events that resulted in significant disability or incapacity;
  any important medical events that might not be immediately life threatening or that resulted in death or hospitalization, but might jeopardize the patient or require intervention to prevent one of the above outcomes;
  any adverse events that required discontinuation of medication;
  thromboembolic events.

The secondary outcome measures were:

reduction of bleeding complications assessed by any scale;
improvement in coagulation status assessed by any scale;
recurrence of bleeding after the surgery;
bleeding during the surgery;
length of hospitalization.

### Search Methods to Identify Studies

#### Electronic searches

Relevant randomized trials were identified by searching The Cochrane Central Register of Controlled Trials (CENTRAL) in The Cochrane Library, MEDLINE, EMBASE, and Science Citation Index, following a structured strategy (see search strategy in Table S1).

### Searching Other Resources

The references of all identified studies were inspected for more trials.

### Selection of Studies

Two authors independently inspected each identified reference and applied the inclusion criteria. For potentially relevant articles or in cases of disagreement between the two reviewers, the full-text article was obtained and inspected independently. If the disagreement could not be resolved by discussion, a third reviewer appraised the article to resolve the disagreement. Justification for exclusion of each study was documented.

### Data Extraction and Management

Two authors independently extracted the data from the included trials. In case of disagreement, a third author extracted the data. The extracted data were discussed and decisions were documented; when necessary, the authors of the original studies were contacted for clarification. Justification for exclusion of each study was recorded. Trials were identified using the last name of the first author and the year in which the trial was published, and these were organized chronologically.

### Characteristics of Studies

The following characteristics of the studies were recorded:

date, location, and setting;
publication status;
case definitions used;
sponsor of trial.

### Characteristics of Participants

The following characteristics of the participants were recorded:

total number of participants;
age, sex, and nationality;
severity of liver disease (by biochemical or radiological data), regardless of the criteria used;
previous treatment with rFVIIa and recent surgical interventions.

### Characteristics of Interventions

The following characteristics of the interventions were recorded:

usage of prophylactic rFVIIa: dose, mode of administration, schedule, and length of follow-up (months);
characteristics of hepatic resection, liver transplantation, and indications;
other interventions to prevent bleeding.

### Assessment of Risk of Bias in Included Studies

Two authors independently assessed the bias risk of the trials without masking the trial names. For this purpose, instructions given in the Cochrane Handbook for Systematic Reviews of Interventions [7] were followed.

### Measures of Treatment Effect and Data Analysis

RevMan Analyses (The Nordic Cochrane Centre, Copenhagen, Denmark) was used to analyze the data. Dichotomous data were analyzed by calculating odds ratios (ORs) and 95% confidence intervals (CIs). Continuous data were analyzed calculating mean differences (MDs) and 95% CIs between intervention groups in each trial. Interventions were compared and stratified by the type of surgical procedure (liver resection or OLT).

To analyze differences in red blood cell requirements, data were harmonized across studies by estimating the means and standard deviations from the reported medians and ranges according to the method of Pudar Hozo *et al.* [8]. After harmonization, the MDs were calculated between intervention groups.

### Assessment of Heterogeneity

We checked the heterogeneity of effects across trials by visual inspection of the forest plots and χ^2^ and *I* ^2^ tests for heterogeneity [7]. Statistical heterogeneity was defined as P>0.10 (χ^2^) or *I* ^2^>25%. Potential sources of heterogeneity were assessed by stratification, mainly, type of surgical procedure (liver resection or OLT) and high and low rFVIIa doses.

### Sensitivity Analysis

We analyzed the data using both fixed- and random-effects models. When both models produced similar estimates, the fixed-effect result was reported. Outcomes were analyzed as reported in the trial; that is, either per protocol or as intention-to-treat analysis.

### Assessment of Reporting Biases

A funnel plot estimating the precision of trials (plot of the logarithm of the relative risk against the sample size) was used to detect publication bias. In addition, the standard normal deviate (SND), defined as the relative risk divided by its standard error, was regressed against the estimate's precision (regression equation: SND = a+b×precision) to facilitate the prediction of potential heterogeneity or data irregularities in the meta-analyses [9]. In this equation, the SND reflects the degree of funnel plot asymmetry as measured by the intercept from the regression analysis.

## Results

### Study Selection

**Figure 1:**
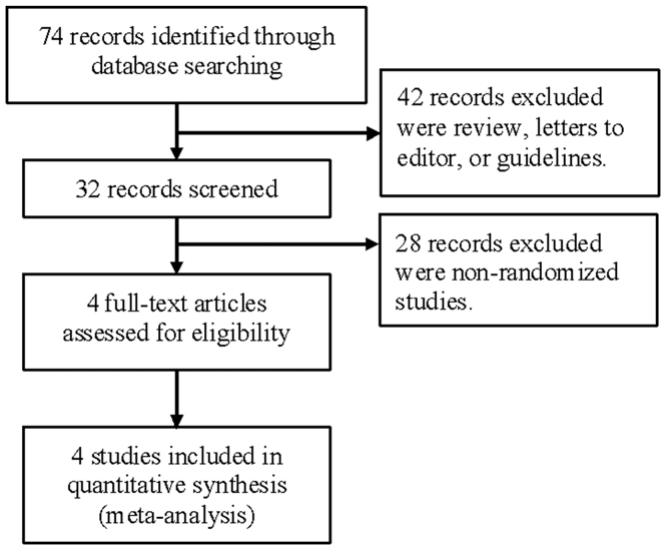
Flow diagram for trial selection.

A total of 74 potential references were retrieved and screened: 31 were narrative reviews or letters to the editor, 28 were nonrandomized studies, 10 were systematic reviews or meta-analyses of different target trials, and one was a clinical guideline. Finally, four randomized controlled trials were included in the analysis (Figure 1).

### Study Characteristics

We included four randomized controlled trials designed to evaluate rFVIIa prophylaxis in liver surgery. The number of patients included in the trials who underwent partial hepatectomy [3], [10] or OLT [4], [11] ranged from 83 to 221. The countries involved were Spain, Germany, France, United Kingdom, China, Taiwan, and Thailand. All but one trial involved cirrhotic patients [3]. The doses of rFVIIa ranged from 20 to 120 µg/kg; although different, all rFVIIa prophylactic schemes were compared against placebo. In the study by Lodge *et al.* [3], a presurgical dose was used and another dose was administered if surgery lasted more than 6 hours. In the studies by Shao *et al.* [10] and Lodge *et al.* [11], a presurgical dose was used with additional doses every 2 hours. In the study by Planinsic *et al.* [4], a single presurgical dose was used. The study by Shao *et al.* [10] evaluated the efficacy of rFVIIa in reducing red blood cell transfusions in patients undergoing partial hepatectomy. Lodge *et al.* [3] evaluated the hemostatic effect and safety of rFVIIa in patients receiving a major partial hepatectomy. Planinsic [4] and Lodge *et al.* [11] evaluated the efficacy and safety of rFVIIa in reducing bleeding and transfusion requirements in patients receiving OLT. All studies were randomized, double-blind, placebo-controlled trials (Table 1).

**Table 1:** Characteristics of the included studies.

	Lodge 2005a	Shao 2006	Lodge 2005	Planinsic 2005
Population (patients)	185 (PH)	221 (PH)	182 (OLT)	83 (OLT)
Intervention	rFVIIa 20, 80 µg/Kg	rFVIIa 50, 100 µg/Kg	rFVIIa 60, 120 µg/Kg	rFVIIa 20, 40, 80 µg/Kg
Comparison	Placebo	Placebo	Placebo	Placebo
Outcome	Haemostatic effect and safety of rFVIIa	Efficacy of rFVIIa in reducing blood transfusions	Efficacy and safety of rFVIIa in reducing transfusion requirements	Efficacy and safety of rFVIIa in the reduction of bleeding in OLT
Study	Randomized, double blind, placebo controlled trial	Randomized, double blind, placebo controlled trial	Randomized, double blind, placebo controlled trial	Randomized, double blind, placebo controlled trial

PH: Partial hepatectomy; OLT: Orthotopic liver transplant; rFVIIa: Recombinant activated coagulation factor VII.

### Risk of Bias within Studies

All trials included were at risk of bias. Sequence generation and allocation concealment were not described properly and remained unclear for evaluation purposes. Another important source of bias was sponsorship because all studies were sponsored by the pharmaceutical industry (Table 2).

**Table 2:** Risk of bias assessment of the included trials.

Source of bias	Lodge 2005	Lodge 2005a	Planinsic 2005	Shao 2006
Sequence Generation	Unclear	Low risk of bias	Low risk of bias	Unclear
Allocation concealment and blinding	Unclear	Low risk of bias	Unclear	Unclear
Incomplete outcome data	Low risk of bias	Low risk of bias	Low risk of bias	Low risk of bias
Selective outcome reporting	Low risk of bias	Low risk of bias	Low risk of bias	Low risk of bias
Other sources of Bias*	High risk of bias	High risk of bias	High risk of bias	High risk of bias

*The other source of bias in all trial was the source of the financial support (industry).

### Synthesis of Results

#### Mortality

**Figure 2:**
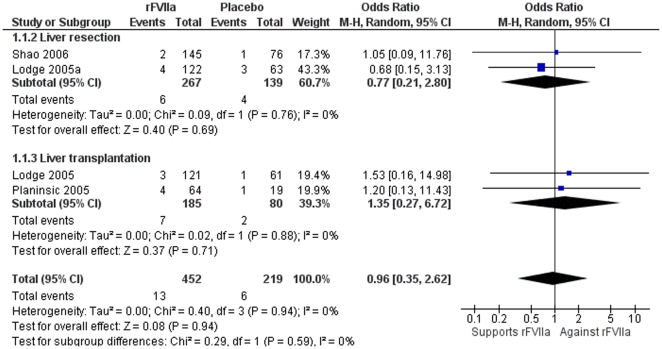
Meta-analysis plot for mortality after liver resection and liver transplantation. There were no differences overall or according to the type of hepatobiliary surgery.

The four trials included assessed mortality and involved 671 subjects. The mortality rate did not differ significantly (OR 0.96; 95% CI 0.35–2.63). There were also no significant differences in mortality stratified according to the type of surgery into liver transplantation (OR, 1.35; 95% CI 0.27–6.72) compared with liver resection (OR 0.77; 95% CI 0.21–2.80) (Figure 2) or according to rFVIIa dose of ≤40 µg/kg (OR 1.36; 95% CI 0.38–4.93) compared with >40 µg/kg (OR 0.67; 95% CI 0.21–2.13) (data not shown).

#### Red blood cells requirement

**Figure 3:**
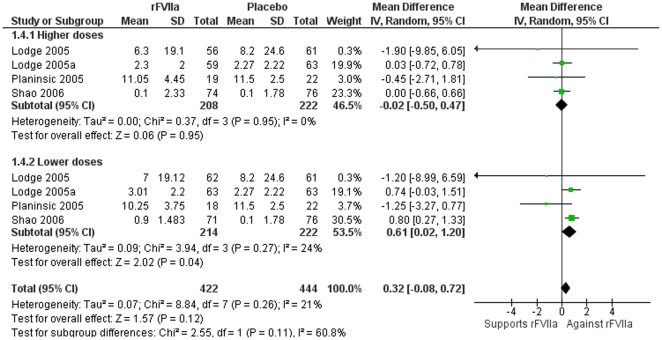
Meta-analysis plot for the number of blood cell units transfused according to the rFVIIa dose. The dose was classified as lower or higher. Slightly more units were transfused in the low-dose rFVIIa group compared to the placebo group (marginal effect).

In the trial by Lodge *et al.* [11], the requirement for red blood cells was reduced from 8.2 units in the placebo group to 7 units in the 60 µg/kg rFVIIa group and to 6.3 units in the 120 µg/kg group (Table 3). However, no differences in the requirement for red blood cells were observed in the other studies. Overall, the data did not show significant benefits in terms of the mean number of blood cell units transfused (MD 0.32; 95% CI −0.08–0.72 units), although a marginal effect was observed in the lower dose groups (MD 0.61; 95% CI 0.02–1.20 units) (Figure 3).

**Table 3:** Secondary outcomes assessed in the included trials.

	Units of FFP(median)	Units of RBC(median)	Hospitalization length (days)(average)
Lodge 2005			
Placebo	11	8.2	17
60 µg/Kg	9.4	7	19
120 µg/Kg	11.9	6.3	22
Planinsic 2005			
Placebo	11	8	
20 µg/Kg	8.5	8.5	
40 µg/Kg	15.5	13	
80 µg/Kg	6	7	
Lodge 2005a			
Placebo			7
20 µg/Kg			7
80 µg/Kg			7
Shao 2006			
Placebo	0	0	
50 µg/Kg	0	0	
100 µg/Kg	0	0	

#### Serious adverse events

**Figure 4:**
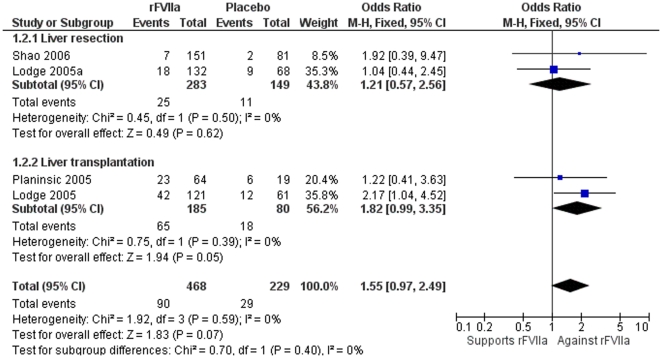
Meta-analysis plot for serious adverse events in liver resection and liver transplantation. There were no differences for liver resection and overall, but a trend towards an increasing serious adverse event rate was observed in the liver transplantation group.

The overall rate of serious adverse events did not differ significantly (OR 1.55; 95% CI 0.97–2.49). There were no differences in the sensitivity analysis between surgical procedures of liver transplantation (OR 1.82; 95% CI, 0.99–3.35) compared with liver resection (OR 1.21; 95% CI 0.57–2.56) (Figure 4), or according to the dose of rFVIIa≤40 µg/kg (OR 1.27; 95% CI 0.61–2.64) compared with >40 µg/kg (OR 1.29; 95% CI 0.77–2.16) (data not shown).

#### Thromboembolic events

**Figure 5:**
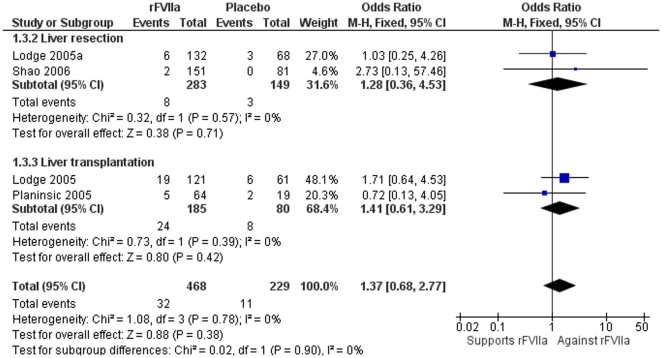
Meta-analysis plot for thromboembolic events in liver resection and liver transplantation. There were no differences overall or by type of hepatobiliary surgery.

The rate of thromboembolic events did not differ significantly (OR 1.37; 95% CI 0.68–2.77). Sensitivity analysis did not show differences in thrombolytic events between liver transplantation (OR 1.41; 95% CI 0.61–3.29) and liver resection (OR 1.28; 95% CI 0.36–4.53) (Figure 5), or between rFVIIa dose ≤40 µg/kg (OR 0.85; 95% CI 0.25–2.94) and >40 µg/kg (OR 1.37; 95% CI 0.68–2.77) (data not shown). The thromboembolic events reported were acute myocardial infarction in three trials (four cases in the rFVIIa group [3], [12], [13]), portal vein thrombosis in two trials (one patient in the placebo [3] and one in the rFVIIa group [13]), and pulmonary embolism in two trials (two patients in the rFVIIa group [3] and one in the placebo group [13]). Other thromboembolic events were suspected to be mesenteric vein thrombosis [13], arterial thrombosis, and thrombophlebitis [12].

#### Secondary outcomes

There was great heterogeneity regarding secondary outcomes. Lodge *et al.* did not observe differences in the duration of hospitalization between groups (17, 19, and 22 days for placebo, 60 µg/kg, and 120 µg/kg, respectively) [11]. Shao *et al.* found no changes in the surgical time from rFVIIa prophylaxis (152, 167, and 144 minutes for placebo, 50 µg/kg, and 100 µg/kg, respectively). Lodge *et al.* [3] found similar results (4.06, 4.04, and 3.61 hours for placebo, 20 µg/kg, and 50 µg/kg, respectively). Lodge *et al.* [3] found no reduction in bleeding during surgery (1422, 1372, and 1073 ml for placebo, 20 µg/kg, and 50 µg/kg, respectively).

## Discussion

FVIIa was developed in 1985 and was introduced to treat patients with hemophilia in 1989 [14]. Good outcomes were observed in these patients, which led to the promotion of rFVIIa use in other settings. There is some evidence about the safety and efficacy of rFVIIa for controlling hemorrhage in patients without hemophilia, mainly in pediatric populations [15]. In adults, hemorrhagic obstetric complications can be reduced by rFVIIa [16], and patients with acute intracerebral hemorrhage may also benefit from the treatment [17]. In patients with trauma and coagulopathy, rFVIIa reduces fresh-frozen plasma transfusions, multiorgan failure, and acute respiratory distress syndrome [18]. Prophylactic use of rFVIIa has been tested in other surgical fields, and several randomized clinical trials have suggested that its use reduces the number of required blood cell transfusions without significantly affecting mortality [19], [20], [21].

In the field of liver diseases, in which impaired coagulation is almost the rule [22], rFVIIa might provide an adequate approach for preventing bleeding complications. The most important surgical procedures in these patients are liver transplantation and liver resection. Noncontrolled studies suggested that rFVIIa significantly reduces mortality [23], [24] without increasing serious adverse events [25]. However, to our knowledge, no systematic review or meta-analysis has assessed the role of rFVIIa in bleeding prophylaxis in liver transplantation or liver resection.

In this meta-analysis, we included four randomized controlled trials, three of them in patients with cirrhosis. Use of prophylactic rFVIIa did not significantly modify the rates of mortality and serious adverse events. Prophylactic rVIIa failed to show a consistent improvement in proximal benefit indicators, such as the number of blood transfusions, although a marginal reduction was observed in the low-dose group. An interesting yet unexpected trend towards more serious adverse events among patients undergoing liver transplantation who were treated with rFVIIa was observed. The intervention did not increase the risk for thromboembolic events. An increased risk has been reported elsewhere, although this observation could reflect the specific characteristic of the clinical setting [17], [26].

The present meta-analysis included studies of the effectiveness of prophylactic rVIIa given to patients undergoing either hepatic transplantation or liver resection. Transplantation patients are at increased risk of mortality and morbidity because of immunosuppression or graft rejection. However, these procedures share hepatic resection and large bleeding as a common characteristic, suggesting that they might also benefit from prophylactic rFVIIa. We observed a homogeneous lack of effect of rFVIIa prophylaxis in either surgical group for all outcomes studied.

The present meta-analysis is limited by the small number of trials included, precluding any definite conclusions about the value of the prophylactic use of rFVIIa in hepatobiliary surgery. Red blood cells requirement was the primary outcome in all studies included, under the expectation that this indicator would be heavily influenced by prophylactic rFVIIa. However, differences in the reporting of transfused blood units precluded a direct comparison between studies. A mathematical transformation was used to generate a global estimate of the effect of rFVIIa over the adjusted number of transfused blood units; while scientifically sound, this transformation may not necessarily reflect the real effect accurately. Fortunately, all studies provided comparable mortality data, allowing a fair comparison of this more distal but important outcome. Such an approach assumes that a reduction in mortality should reflect an improvement of intermediate variables. We did not observe such association. Still, readers should exert caution while interpreting mortality results, since the original studies were not powered to specifically evaluate this outcome.

Considering all available evidence together, prophylactic rFVIIa failed to significantly modify any of the outcomes studied. This has implications for clinical practice because there appears to be no clear evidence to promote its use in OLT or liver resection. In addition, no high-quality trials have been published since 2006, and no additional trials have been registered in ClinicalTrials.gov. More trials are needed to adequately evaluate the use of prophylactic rFVIIa in liver resection and transplantation.

## Supporting Information

Table S1 (DOC)
