# Supplementary material for: Prophylactic Activated Recombinant Factor VII in Liver Resection and Liver Transplantation: Systematic Review and Meta-Analysis
Source: PLoS One. 2011 Jul 27;6(7):e22581. doi: 10.1371/journal.pone.0022581 (PMC3144913; doi:10.1371/journal.pone.0022581)
Supplement: Table S1 — (DOC) [file pone.0022581.s001.doc]

The Cochrane Hepato-Biliary Group Controlled Trials Register (December 2010)

(novoseven OR Recombinant activated coagulation factor VII) AND (hepat* OR liver) AND (surg* OR hepatectom* OR transplant*)

MEDLINE 1950-Dec 2010

#1 explode Factor VIIa /All Subheadings

#2 recombinant FVIIa

#3 rFVIIa

#4 recombinant factor VIIa

#5 eptacog alfa

#6 Novo Seven

#7 novoseven

#8 #1 OR #2 OR #3 OR #4 OR #5 OR #6 OR #7

#9 explode liver /All subheadings

#11 hepatic

#12 hepat*

#13 #10 OR #11 OR #12

#14 explode general surgery /All subheadings

#15 explode liver transplantation /All subheadings

#16 explode hepatectomy /All subheadings

#17 #14 OR #15 OR #16

#18 random* or blind* or placebo* or meta-analysis

#19 #8 AND #13 AND #17

CSI

TS=(Factor VIIa OR eptacog alfa OR Novo Seven OR novoseven)

TS=(liver OR hepat*)

TS=(liver OR hepat*)

TS=(random* or blind* or placebo* or meta-analysis)
